# Primary Lymphoepithelioma-Like Carcinoma of the Conjunctiva Metastatic to Regional Lymph Nodes and Parotid Gland in a Mexican Patient

**DOI:** 10.1155/2022/9265244

**Published:** 2022-02-14

**Authors:** Ana Lía Díazceballos-García, Sebastian Diener-Kudisch, Abelardo A. Rodriguez Reyes, Ángel Nava-Castañeda

**Affiliations:** ^1^Oculoplastics Department, Instituto de Oftalmología Fundación Conde de Valenciana, Mexico City, Mexico; ^2^Ophthalmic Pathology Service, Asociación para Evitar la Ceguera en México, I. A. P., Mexico City, Mexico

## Abstract

Lymphoepithelioma-like carcinoma (LELC) of the conjunctiva is a rare malignancy in the ocular adnexa. There are no prospective data regarding treatment methods. Complete surgical excision is sufficient in the majority of cases. Radiation therapy is sometimes used in case of recurrence or positive margins after surgery. This case describes an 89-year-old Hispanic female with a 7-month history tumor primarily located on the left lower palpebral conjunctiva. The patient underwent an excisional biopsy of the tumor, and histopathology exam reported an LELC with positive margins. She developed parotid and neck lymph node metastasis treated with concurrent radiotherapy. The patient had remained disease-free for 3 years. According to the available data, there are only five cases of LELC reported in conjunctiva worldwide, so this report increases the differential diagnoses of tumors in the ocular adnexa and supports the effectiveness of radiotherapy.

## 1. Introduction

Lymphoepithelioma-like carcinoma (LELC) is a rare cutaneous malignant neoplasia first described in 1988 by Swanson et al. It presents itself as a plaque-like, firm, variously colored, facial or neck nodule and affects mostly Asian and elderly population, with similar incidence in men and women [1]. Although its etiology is mostly unknown, it is believed that it has an adnexal and epidermic origin, based on eccrine and trichilemmal differentiation [2]. It consists of uniform tumor cells, with moderate amount of eosinophilic cytoplasm and vesicular nuclei with one or two prominent nucleoli that form multiple nodules, smaller irregular islands, and cords associated with a lymphoid infiltrate. Although it may resemble nasopharyngeal lymphoepithelioma, other locations of LELC can be the stomach, salivary gland, lung, thymus, skin, uterine cervix, tonsil, oral cavity, trachea, larynx, urinary bladder, and vagina [3]. Like other lymphoid cell malignancies, this neoplasm has been associated in some tissues with the Epstein-Barr virus (EBV) in certain populations; nevertheless, this has only been reported in the Asian population for the orbit and ocular adnexa [3–5]. Treatment of this neoplasm may include surgery, radiotherapy, or chemotherapy [4, 6, 7]. This neoplasm represents a significant diagnostic challenge because it is an extremely rare presentation as a primary tumor in the orbit, eyelids, and ocular adnexa. We described a case of a primary lymphoepithelioma-like carcinoma of the palpebral conjunctiva showing parotid and neck lymph node metastasis. This report is compliant with the Health Information Portability and Accountability Act and the tenets of the Declaration of Helsinki.

## 2. Case Report

**Figure 1:**
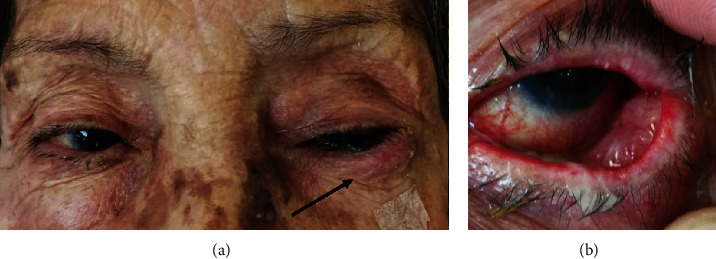
(a) Photograph showing a tumor located in the lower external half of the left palpebral conjunctiva. (b) A palpable 25 × 15 mm, nonpainful, pinkish, poorly mobile, smooth-surfaced, dome-shaped elevated tumor causing slightly lid eversion was appreciated in the external third of the left lower palpebral conjunctiva.

An 89-year-old female Mexican patient was admitted to “Instituto de Oftalmología Fundación Conde de Valenciana” with a 7-month history of an erythematous, nontender, nonpainful lesion in the left eyelid. The patient had right eye (RE) cataract surgery five years ago. She also had a history of an ocular surface squamous neoplasia (OSSN) in the left eye successfully treated with topical interferon alfa 2b five years ago and a chalazion in the lower left eyelid diagnosed at a local clinic 7 months ago treated with oral and local antibiotic therapy, which was ineffective. Upon ophthalmologic examination, the best-corrected visual acuity was 20/30 in the RE and 20/100 in the LE. Both eyes showed no motility disturbances, pupils were normal, and no diplopia was reported by the patient. Intraocular pressures were 13 mmHg and 14 mmHg on the RE and LE, respectively. Anterior segment showed a senile cataract on the LE, and fundoscopic examination showed small drusen in both eyes. On slit lamp examination, a palpable 25 × 15 mm, pinkish, poorly mobile, smooth-surfaced, dome-shaped elevated tumor causing slightly lid eversion was detected at the external third of the left lower palpebral conjunctiva. There was no loss of eyelashes associated with the neoplasm, no palpable regional lymphadenopathies were found at this point, and systemic examination was unremarkable (Figures 1(a) and 1(b)).

**Figure 2:**
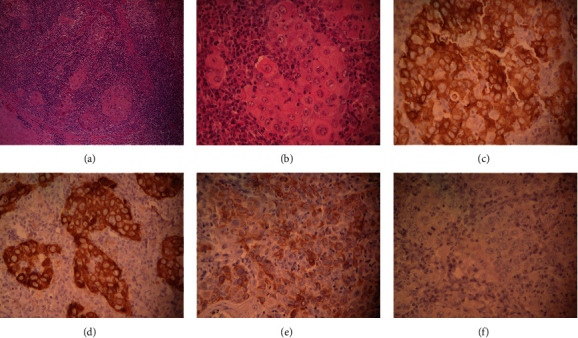
Photomicrographs of histopathological and immunohistochemical findings. (a, b) Different portions of the eyelid are involved in their entire thickness, by a malignant neoplasm composed of sheets, syncytia, and solid nests of cells with epithelioid appearance with moderate amount of eosinophilic cytoplasm with poorly differentiated borders and round to oval vesicular nuclei with one or two nucleoli and numerous typical and atypical mitoses. The nests of neoplastic cells are intermingled with areas of coagulative necrosis and surrounded by inflammatory infiltrate composed of lymphocytes and plasma cells. The superficial epithelium is ulcerated in the majority of its extent (H&E, original magnification 10x and 40x). (c–f) Immunohistochemistry with primary antibodies against epithelial membrane antigen (EMA), pan-cytokeratin AE1/AE3, and cytokeratin 5/6 (CK5/6) was positive for neoplastic cells. Latent membrane protein 1 (PML-1) was negative (ruling out association with EBV infection) as well as HMB45 (ruling out melanoma).

**Figure 3:**
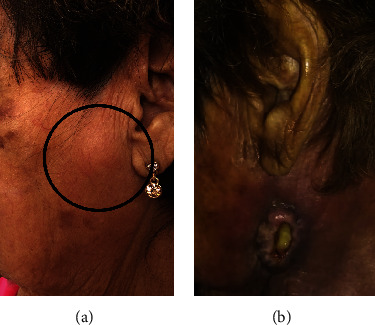
(a) Left preauricular lymph node. (b) Neck with lymph node metastasis.

The patient underwent wide surgical excision, and the residual eyelid defect was reconstructed with a tarsoconjunctival tarsal flap. On pathological examination, the tumor revealed a malignant neoplasm formed by sheets, syncytia, and solid nests of cells with epithelioid appearance, surrounded by a dense lymphoplasmacytic infiltrate. A representative microphotograph of a LELC was provided (Figures 2(a) and 2(b)). To support LELC diagnosis, immunohistochemistry for epithelial membrane antigen (EMA), pan-cytokeratin AE1/AE3, and cytokeratin 5/6 (CK5/6) was performed showing positive staining for neoplastic cells. The latent membrane protein 1 (LMP-1) immunoreaction was negative for neoplastic cells, ruling out Epstein-Barr virus infection association. The HMB45 immunoreaction was negative to neoplastic cells, discarding melanoma-like neoplasia (Figures 2(c)–2(f)). Upon microscopic examination, the medial and temporal eyelid margins were found to be positive. Unfortunately, the patient was lost to follow-up. She returned to the clinic 3 months later presenting a swollen, nonpainful left preauricular lymph node (Figure 3(a)). No macroscopic lesion was perceived in the surgical area of the left eyelid at that moment. The patient was referred to an oncologic medical center where a metastatic workup, including head, orbit, and maxillofacial computed tomography (CT) scans and preauricular, submandibular, and laterocervical lymph node ultrasonography, was performed. Adjuvant radiotherapy was indicated. She received 80 Gy in 35 fractions to the orbit and metastatic lymph nodes. One year later after primary diagnosis, parotid and neck lymph node regional invasion was reported (Figure 3(b)). Palliative radiotherapy with 37.5 Gy was given in 15 fractions. At the time of this report, 3 years have gone by; the patient is still alive and she has been followed up with annual ophthalmic examination and currently monitored for other sites of metastasis.

## 3. Discussion

This case displays a primary conjunctival LELC showing regional lymph node and parotid gland metastasis showing good response to adjuvant radiotherapy. LELC of ocular adnexa is infrequent, only 22 case reports and case series exist in current literature, and 5 of those originate from the conjunctiva [4, 8]. The most common presentation of this neoplasm is in the Asian population, and this case is described in a Mexican patient, rendering it furthermore relevant. The most common age of presentation of LELC in ocular adnexa ranged from 45 to 95 years (median: 66 years); the age of our patient at the time of diagnosis was 89 years concurring with the range of age described. The clinical manifestations of ocular adnexal LELC varied from site to site: diplopia and proptosis were commonly seen in lacrimal gland tumor; epiphora was a common symptom in lacrimal sac tumor; rhinorrhea and epistaxis were seen in nasolacrimal duct tumor; and a palpable mass was usually the only manifestation in conjunctiva or eyelid tumor [4, 8]. The only complaint of the patient of the present report was a mass inside the eyelid. This mass was incorrectly diagnosed as a chalazion that did not improve with medical treatment, similar to the patient described by Kim et al. [9].

The histopathology of LELC is characterized by nests, cords, or sheets of mitotically active polygonal epithelioid cells with scant amphophilic to eosinophilic cytoplasm, hyperchromatic nuclei, coarse chromatin granules, and 1 or 2 prominent nucleoli [6]. The aforementioned description matches to what was found in the present case: the tumor presented in their entire thickness a malignant neoplasm composed of sheets, syncytia, and solid nests of cells with epithelioid appearance with moderate amount of eosinophilic cytoplasm with poorly differentiated borders and round to oval vesicular nuclei with one or two nucleoli and numerous typical and atypical mitoses. The association between EBV and LELC varies by site and by patient ethnicity. Infection with EBV is associated with LELC of salivary gland and lung in Asian patients. It is also associated with LELC of the stomach and thymus independent of ethnicity. The association between EBV and LELC in the ocular adnexa seems to be restricted to Asian populations [4]. Accordingly, the Mexican patient of this report presented a negative latent membrane protein 1 (LMP-1) immunoreaction in neoplastic cells, ruling out the association with EBV infection. Negative tests to EBV have been reported in non-Asian population [5]; however, further cases are needed to establish a clear relationship between EBV and LELC in Latin American population.

As Maruyama et al. described [10], our differential diagnosis included sebaceous carcinoma, squamous cell carcinoma, malignant melanoma, Merkel cell carcinoma, lymphoproliferative condition, mesenchymal tumor, and carcinoma metastasis. The tumor in the present case did not show tendencies towards keratinization or sebaceous differentiation. In malignant melanoma, tumor cells should contain melanosomes; these were not found in the present tumor. Besides, HMB45 immunoreaction was negative in neoplastic cells, ruling out melanoma. In Merkel cell carcinoma, neuroendocrine granules and dense core granules are present and the tumor cell is smaller; the nuclear-cytoplasmic ratio is higher than in LELC. Carcinoma metastasis should show some evidence of a primary lesion elsewhere.

Ancillary tests like computed tomography (CT) scan and/or magnetic resonance imaging (MRI) have been proven to be very helpful to determine the LELC tumor characteristics and its extension, location, and invasion to local structures. The CT scan reports on LELC ocular adnexa mostly describe a homogenous, well-defined soft-tissue mass in the affected area [4]. In the present case, auxiliary tests were not performed because we considered that the tumor was well located in the palpebral conjunctiva without local invasion. Nevertheless, we strongly recommend to perform image study before surgery to discard invasion to adjacent structures and to design the best surgery approach.

Since LELC is such a rare neoplasm, there are no prospective data on treatment methods. Only series of cases and case reports have been published in the literature so far. Surgery alone is sufficient in the majority of cases; however, especially in cases with positive margins, as presented in the present case, or perineural invasion, adjuvant therapy is recommended. Such treatment should be started immediately after wound healing. Unfortunately, in our case, the patient was lost in her follow-up, and when she returned to her ophthalmic surgery control, she had already developed a preauricular lymph node strongly suggesting regional metastasis. LELC of the head and neck is reported to be sensitive to radiotherapy, with good locoregional control. Dubey et al. [11] studied 34 patients with lymphoepithelioma of the head and neck, being the main site of the tumor in the oropharynx (24 patients). Treatment consisted of radiotherapy for 24 patients, excisional biopsy of the primary tumor followed by radiotherapy for 7 patients, and surgery for 3 patients. They reported that for the irradiated patients, the 5-year regional control rates were 77% overall and 83% within the radiation field. In the ocular adnexa, the disease also seems to be radiosensitive [4]. Prior radiation dosing strategies specifically for conjunctival LELC include the administration of radiotherapy of 50 Gy in 25 fractions [12]. In our patient, adjuvant radiotherapy achieved remission of locally residual tumor and parotid and neck lymph node metastasis similar to the case reported by Mucha-Małecka et al. [7]. Survival in ocular adnexal LELC varies by site and histology. Qiu et al. [4] reported a survival rate between 22 and 76 months after surgery. Because of the small number of cases and short follow-up, the overall survival in ocular adnexal LELC is difficult to assess. Qi et al. [13] studied a total of 2106 LELC patients and reported that the median cancer-specific survival (CSS) according to primary tumor site was 90 months for nasopharyngeal LELC and 77 months for nonnasopharyngeal head and neck LELC. The CSS at 5 years for nasopharyngeal LELC was 74%, which was significantly worse than nonnasopharyngeal head and neck LELC (5-year CSS: 85%). They concluded that the prognosis of nonnasopharyngeal head and neck LELC is significantly better than that of nasopharyngeal LELC.

To conclude, LELC is a rare and unpredictable neoplasm regarding its location and evolution. For early and locoregionally advanced disease, complete excision of tumor plus postsurgical radiotherapy seems to achieve satisfactory disease control. Because of the low incidence of this entity, further investigation is needed to determine the optimal treatment and prognosis. Further efforts with a larger number of cases and longer-term follow-up are necessary.

## Data Availability

The photos, composite figures, and histological and auxiliary studies to support the findings of this case report may be released upon written application to the Photographic, Laboratory, and Clinical Archives Department at Instituto de Oftalmología Fundación Conde de Valenciana (nonprofit organization), Chimalpopoca 14, Colonia Obrera, Mexico City 06800, Mexico, and from the corresponding author upon request.
